# Thioredoxin *o*-mediated reduction of mitochondrial alternative oxidase in the thermogenic skunk cabbage *Symplocarpus renifolius*

**DOI:** 10.1093/jb/mvy082

**Published:** 2018-10-05

**Authors:** Yui Umekawa, Kikukatsu Ito

**Affiliations:** 1 Cryobiofrontier Research Center, Faculty of Agriculture, Iwate University, 3-18-8 Ueda, Morioka, Iwate, Japan; 2 Department of Biological Chemistry and Food Science, Faculty of Agriculture, Iwate University, 3-18-8 Ueda, Morioka, Iwate, Japan; 3 Agri-Innovation Research Center, Iwate University, 3-18-8 Ueda, Morioka, Iwate, Japan

**Keywords:** AOX, mitochondria, NADPH, redox regulation, thermogenesis

## Abstract

Thermogenesis in plants involves significant increases in their cyanide-resistant mitochondrial alternative oxidase (AOX) capacity. Because AOX is a non-proton-motive ubiquinol oxidase, the dramatic drop in free energy between ubiquinol and oxygen is dissipated as heat. In the thermogenic skunk cabbage (*Symplocarpus renifolius*), *SrAOX* is specifically expressed in the florets. Although SrAOX harbours conserved cysteine residues, the details of the mechanisms underlying its redox regulation are poorly understood. In our present study, the two mitochondrial thioredoxin *o* cDNAs *SrTrxo1* and *SrTrxo2*, were isolated from the thermogenic florets of *S. renifolius.* The deduced amino acid sequences of the protein products revealed that SrTrx*o*2 specifically lacks the region corresponding to the α3-helix in SrTrx*o*1. Expression analysis of thermogenic and non-thermogenic *S. renifolius* tissues indicated that the *SrTrxo1* and *SrAOX* transcripts are predominantly expressed together in thermogenic florets, whereas *SrTrxo2* transcripts are almost undetectable in any tissue. Finally, functional *in vitro* analysis of recombinant SrTrx*o*1 and mitochondrial membrane fractions of thermogenic florets indicated its reducing activity on SrAOX proteins. Taken together, these results indicate that *SrTrxo1* is likely to play a role in the redox regulation of SrAOX in *S. renifolius* thermogenic florets.

Skunk cabbage (*Symplocarpus renifolius*) is a perennial wildflower that exhibits extensive endogenous thermogenesis during flowering (1). Its spadix is a thermogenic organ that is capable of maintaining its temperature at 23°C as the ambient temperature fluctuates, even in freezing conditions (2). The homeothermic temperature regulation in the spadices is achieved through dynamic respiration control in the thermogenic cells of the tissue (3, 4). Specifically, the respiration rates increase when the spadix temperature shifts from 23°C to lower temperatures, and reach a maximum of 15°C. The temperature at which maximum respiration occurs (15°C) is known as the ‘switching temperature’ (4). Above the switching temperature, the respiration rate is inversely related to temperature in a fully reversible way.

The mitochondria in *S. renifolius* thermogenic spadices have a higher cyanide-insensitive alternative oxidase (AOX) capacity (5)*.* AOX is an integral protein localized on the matrix side of the mitochondrial inner membrane where it catalyses the four-electron oxidation of ubiquinol by oxygen to water (6). Because electron transport by AOX is not coupled with ATP synthesis, the energy of ubiquinol oxidation is released as heat. AOX exists either as a non-covalently associated reduced dimer (active form) or an oxidized dimer (inactive form) through the formation of a disulphide bridge between the conserved cysteine residue Cys I (7, 8). Because the AOX proteins in thermogenic *S. renifolius* florets exist as a reduced active form irrespective of the thermogenic status at different ambient temperature conditions (5), it is hypothesized that a specific mechanism controls the redox status of the AOX protein.

The reduction status of AOX in non-thermogenic plants such as *Arabidopsis*, pea and tobacco have been shown to be regulated via a thioredoxin (Trx) system (9*–*31). The Trxs are small proteins that catalyse thiol-disulphide redox reactions by reversible oxidation of the cysteines in their conserved WCGPC motif at an active site (14). Although plant Trxs are categorized into seven subtypes (*f*-, *m*-, *h*-, *o*-, *x*- *y*-, and *z*-types), and Trx *o* has been shown to reside in plant mitochondria (15), little is known about the molecular mechanisms that control the reduction of mitochondrial AOX in thermogenic plants.

To uncover the possible involvement of Trx in the reduction mechanisms of AOX proteins in thermogenic plants, we here conducted a series of experiments focusing on Trx molecules in *S. renifolius.* We successfully isolated two distinct cDNAs (*SrTrxo1* and *SrTrxo2*) encoding mitochondrial Trx *o* proteins from thermogenic florets of *S. renifolius.* Expression and functional analysis of these *SrTrxo* genes revealed that *SrTrxo1*, but not *SrTrxo2*, plays a role in the redox regulation of AOX protein in *S. renifolius.*

## Materials and Methods

### Plant materials

For isolation of total RNA for cDNA cloning, thermogenic spadices of *S. renifolius* were collected from their natural populations in Fujine (Iwate prefecture, Japan) on 3 April 2012. For preparation of total RNAs for gene expression analysis, florets, piths, leaves, and spathes were sampled from thermogenic *S. renifolius* at Kanegasaki (Iwate prefecture, Japan) on 1 April 2014. For mitochondrial preparation, thermogenic florets of *S. renifolius* from Omori (Akita prefecture, Japan) were sampled on 26 April 2017. The ambient temperatures at each site were 8.3°C (Fujine), 8.2°C (Kanegasaki) and 10.6°C (Omori).

### Molecular cloning of mitochondrial SrTrxo1 and SrTrxo2

Total RNA was extracted from the florets of the thermogenic spadix of *S. renifolius* using NucleoSpin® RNA II (Macherey-Nagel, Düren, Germany) and a Total RNA Extraction Kit Mini (Plant) (RBC Bioscience, New Taipei City, Taiwan). First-strand cDNAs were synthesized using PrimeScript^TM^ II 1st strand cDNA Synthesis Kit (Takara Bio, Shiga, Japan) with an oligo-dT primer. Partial fragments of the targeted transcripts for the *Trx o* gene were amplified by PCR using a primer set listed in Supplementary Table S1. Gene-specific primers (Supplementary Table S1) were designed for 5′- and 3′- RACE using the SMARTer RACE cDNA Amplification Kit (Clontech, Mountain View, CA). Finally, PCR amplification of full-length cDNAs was performed with KOD FX Neo (Toyobo, Osaka, Japan) using the gene-specific primers described in Supplementary Table S1. The obtained products were subsequently cloned into the T-Vector (pMD19; Takara Bio) and sequenced. The full-length cDNA sequences for *SrTrxo1* and *SrTrxo2* were deposited in the DNA Data Bank of Japan with Accession numbers LC107868 and LC107869, respectively. These sequences were analysed using GENETYX software (Genetyx, Tokyo, Japan).

### Prediction of the SrTrxo1 and SrTrxo2 protein structures

Protein structure modelling of SrTrx*o*1 and SrTrx*o*2 was performed with SWISS-MODEL (https://swissmodel.expasy.org/) using the crystal structure of a wild-type poplar, Trx *h* (PtTrxh4; SMTL id: 3d21.1).

### Phylogenetic analyses of Trx proteins

A phylogenic tree of the Trx family proteins was constructed using the neighbour-joining method with ClustalW (http://www.genome.jp/tools-bin/clustalw). The sequences were gathered from GenBank® (Supplementary Table S2). Multiple sequence alignments were performed using MEGA6 for windows as described previously (16).

### Expression analysis of SrTrxo1, SrTrxo2 and SrAOX transcripts

Real-time qPCR was performed with the SYBR Green Real-time PCR Master Mix-Plus (Toyobo) and the THUNDERBIRD® SYBR qPCR Mix (Toyobo) using a Thermal Cycler Dice instrument (TP800; Takara Bio) as described previously (17, 18). Gene-specific primers were designed based on identified gene sequences and are listed in Supplementary Table S1. *Elongation factor 1α* (*EF1α*) was used as a housekeeping gene and normalization control.

### Expression and purification of SrTrxo His-tagged proteins

The ORFs of the mitochondrial targeting sites in *SrTrxo1* and *SrTrxo2* were amplified by PCR using KOD-Plus-Neo (Toyobo) with primers containing *Xho*I sites (underlined) as follows: 5′-AGTTGGCTCGAGAATATTGTGGTTGTTGGC-3′ and 5′-GGAAGGCTCGAGTCATTCCATCTTGTAAAG-3′. The obtained fragments were then cloned into the *Xho*I site of the pET-16b expression vector (Merck Millipore, Darmstadt, Germany), and confirmed by DNA sequencing. BL21 (DE3) cells (ECOS^TM^ competent *E. coli* BL21 (DE3); Nippon Gene, Tokyo, Japan) carrying a pET-16b construct containing *SrTrxo1* or *SrTrxo2* were cultured in LB medium. Expression of the recombinant His-tagged proteins in these cells was induced in the presence of 1 mM IPTG for 3 h at 37°C, and cells were harvested by centrifugation (5000 *g*, for 5 min, 4°C). The pellets were washed in a buffer containing 20 mM Tris–HCl (pH 8.0), and stored at −80°C. The recombinant proteins were subsequently purified from the frozen pellets using His GraviTrap (GE Healthcare, Buckinghamshire, England). Purified proteins were subjected to ultrafiltration with Amicon Ultra-15 Centrifugal Filter Units (Merck Millipore) in a buffer containing 25 mM Tris–HCl (pH 8.1) and 1 mM EDTA.

### Enzyme assay for recombinant SrTrxo1 proteins

Insulin reduction activities of recombinant SrTrx*o*1 proteins were determined using the PROTEOSTAT® Thioredoxin-1 Assay Kit (Enzo Life Sciences, New York) at 15°C, 23°C and 30°C, respectively.

### Purification of mitochondria, preparation of mitochondrial membrane fractions and assay for AOX reduction by recombinant SrTrxo1

Mitochondria were isolated from the florets of *S. renifolius* thermogenic spadices as described previously (19), and stored at −80°C. Frozen mitochondria were deforested, and repeated centrifugations were performed to remove the mitochondrial matrix fraction (5). The resultant mitochondrial membrane fractions were dissolved in a buffer containing 10 mM MOPS-KOH (pH 7.5), 0.3 M mannitol, 2 mM pyruvate, 0.2% [w/v] BSA and cOmpete ULTRA protease inhibitors (Roche, Basel, Switzerland). To assay SrAOX reduction by SrTrx*o*1, a portion of the mitochondrial membrane fraction was incubated in a buffer containing 25 mM Tris–HCl (pH 7.0), 1 mM EDTA, and 1.5 U/ml NADPH-dependent Trx reductase (yeast recombinant NTR; Oriental Yeast, Tokyo, Japan), and 3 µM recombinant SrTrx*o*1 protein (Fig. 6). The level of AOX reduction was examined using non-reducing SDS-PAGE and subsequent western blotting with an AOX polyclonal antibody (Agrisera, Vännäs, Sweden) as described previously (5, 20).

### Statistical analysis

Statistical differences were evaluated by one-way ANOVA with Tukey-Kramer multiple comparison test using the R software (version 3.5.1) (21).

## Results

### The SrTrxo genes encode mitochondrial thioredoxin in S. renifolius

**Fig. 1:**
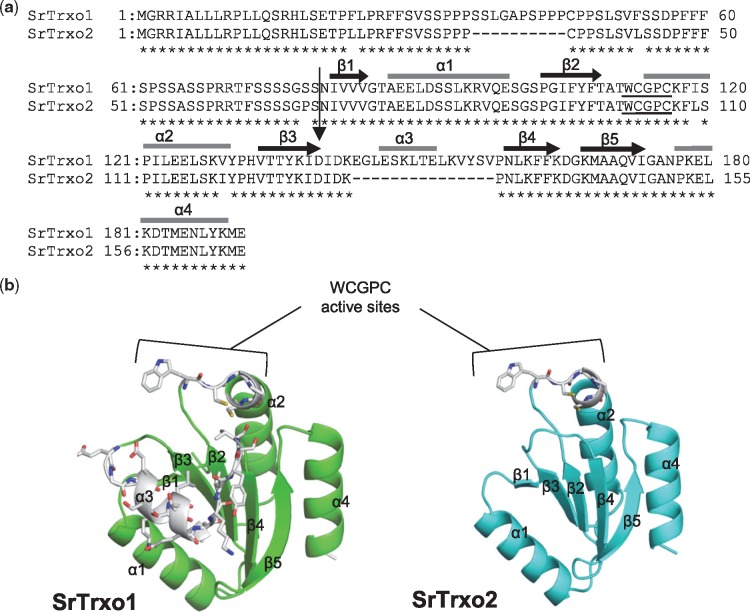
**Deduced amino acid sequences and protein structures of SrTrx*o*1 and SrTrx*o*2**. (**a**) Alignment of the identified sequences of SrTrx*o*1 and SrTrx*o*2. The asterisks indicate the conserved amino acid residues. Underlined sequences denote the WCGPC active sites conserved in the Trx family. An arrow indicates the cleavage site of SrTrx*o*1 and SrTrx*o*2. Potential regions for the α-helix and β-sheet are indicated above the amino acid sequence. (**b**) Predicted protein structures of SrTrx*o*1 and SrTrx*o*2. WCGPC active sites and the position of deleted areas in the genes are indicated by different colours as follows: C (carbon) and H (hydrogen), grey; N (nitrogen), blue; O (oxygen), red; S (sulphur), beige.

**Fig. 2:**
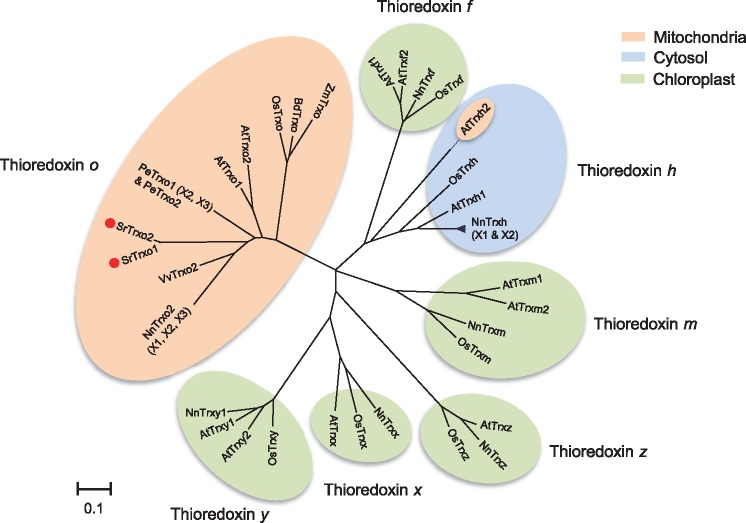
**Phylogenetic tree for the Trx protein family.** A 37 amino acid sequence from the Trx family proteins of several plants was used for phylogenetic tree analysis. The intracellular localization of each Trx isoform is indicated by different colours as follows: mitochondria, orange; cytosol, light blue; chloroplast, light green. SrTrx*o*1 and 2 are denoted by red circles. AtTrx*h*2 is targeted to both mitochondria and cytosol (14*, *40).

cDNAs for *SrTrxo1* and *SrTrxo2*, which each encode a mitochondrial-localized *o*-type Trx, were isolated from *S. renifolius* thermogenic spadices. Mitoprot analysis predicted mitochondrial cleavage sites at the N-terminus of each protein (Fig. 1a). Their deduced amino acid sequences contained WCGPC motifs that are essential for the Trx-mediated reduction of target proteins (22). A further comparison of the putative amino acid sequences and three-dimensional homology models of SrTrx*o*1 and SrTrx*o*2 revealed that SrTrx*o*2 specifically lacked a region that corresponded to the α3-helix in SrTrx*o*1 (Fig. 1b). Phylogenetic analysis of several Trx family proteins, including those from non-thermogenic and thermogenic plants, clearly characterized SrTrx*o*1 and SrTrx*o*2 as *o*-type Trx proteins (Fig. 2).

**Fig. 3:**
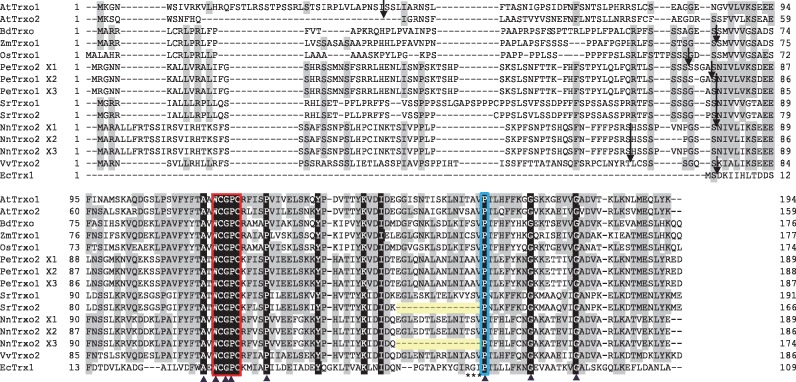
**Alignment of the amino acid sequences of plant Trx *o* and *E. coli* Trx.** The amino acid sequences of several plant Trx *o* proteins are indicated along with that of the Trx from *E. coli.* Arrows indicate the cleavage sites and highly conserved WCGPC active sites and important proline residues are boxed in red and light blue, respectively. Common deletion sequences in SrTrx*o*2 and NnTrx*o*2 X3 are indicated in light yellow. Asterisks indicate the residues that affect the protein dynamics in the reduced Trx from *E. coli* (25)*.*

To further characterize the primary protein structures and conserved domains of SrTrx*o*1 and SrTrx*o*2, multiple amino acid alignments were performed using several plant Trx *o* proteins together with Trx1 from *Escherichia coli.* Two cysteine residues in a catalytic site and a proline residue inside the WCGPC motif that determines the reducing power of Trx (22–24) were conserved across these different Trxs including SrTrx*o*1 and SrTrx*o*2 (Fig. 3). Additionally, conserved residues included a second proline residue proximal to the WCGPC motif that introduces a kink in the α2-helix that separates the WCGPC motif from the rest of the helix (22, 25), and a third proline that is important for both active site conformation and redox potential (22, 26) (Fig. 3). Moreover, three glycine residues and a tryptophan, alanine and lysine residue were also conserved in these proteins across the different plant species and *E. coli* (Fig. 3). A specific deletion of the α3-helix domain in SrTrx*o*2 was also found in the *Nelumbo nucifera* Trx*o*2 (Fig. 3). Taken together, these results indicated that both SrTrx*o*1 and SrTrx*o*2 function as a Trx enzyme.

### Gene expression analysis of SrTrxo1, SrTrxo2 and SrAOX in thermogenic and non-thermogenic S. renifolius tissues

**Fig. 4:**
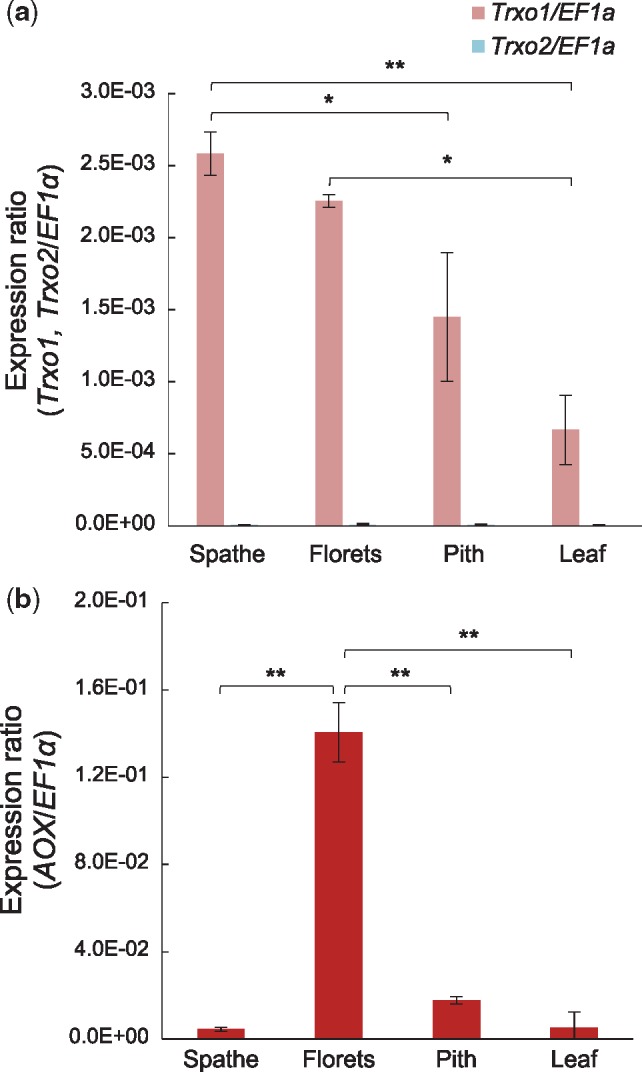
**Expression analysis of *SrTrxo1*, *SrTrxo2* and *SrAOX* transcripts.** Expression levels of *SrTrxo1* and *SrTrxo2* (**a**), and *SrAOX* (**b**) in various tissues of *S. renifolius* during thermogenesis. *SrEF1α* transcripts were used as a normalization control. All experiments were performed in triplicate for each sample. Data are mean ± SD (*n* = 3). Significance of one-way ANOVA with Tukey-Kramer multiple comparison test is indicated as follows: *P < 0.05 and **P < 0.01.

To determine the expression levels of *SrTrxo1* and *SrTrxo2*, we used qRT-PCR analysis of RNA isolated from the florets, pith, leaf and spathe of *S. renifolius* in its thermogenic stage. Previous thermal imaging analysis has found that the florets are the thermogenic tissue whereas the pith, leaf and spathe are non-thermogenic (1, 18). The expression of the *SrAOX* gene has already been shown to reflect thermogenic activity (5), and this gene was thus included in this analysis as a marker of the thermogenic status of the examined *S. renifolius* tissues. The results clearly showed that *SrTrxo1* transcripts are expressed in all tissues, but that the levels are tissue specific. The highest expression detected in florets and spathes, compared with a moderate expression level in the pith, and lowest expression in the leaves (Fig. 4a). In contrast, *SrTrxo2* mRNA was almost undetectable in every tissue tested (Fig. 4a). We found that *SrAOX* is abundantly expressed in the florets (Fig. 4b), indicating that they are indeed thermogenic. Our findings in these analyses suggested that although thermogenic florets have the highest expression of *SrTrxo1*, expression of this gene is not regulated in a thermogenic tissue-specific manner. Our data also suggest that *SrTrxo2* does not contribute to thermogenesis in *S. renifolius.*

### Production of SrTrxo proteins and thioredoxin activity

**Fig. 5:**
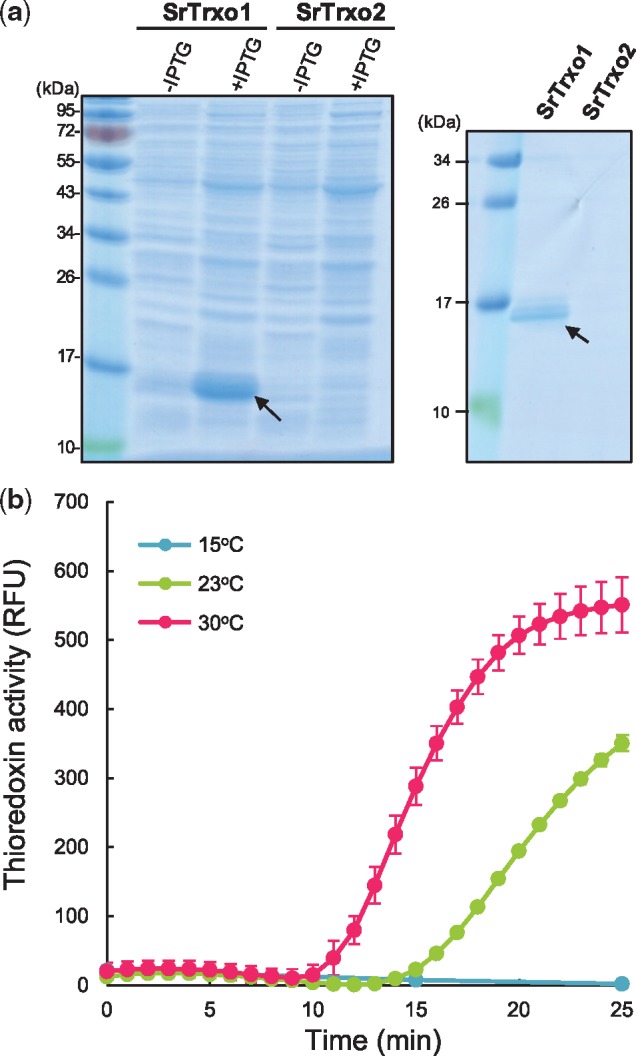
**Preparation, purification and insulin reduction assay of recombinant mitochondrial Trx proteins, SrTrx*o*1 and SrTrx*o*2.** (**a**) Preparation and purification of His-tagged SrTrx*o*1 and SrTrx*o*2 proteins. *SrTrxo1* and *SrTrxo2* genes were each subcloned into a pET-16b vector. Induction of recombinant protein expression was done in the presence of IPTG (1 mM) (left), and subsequent purification using an Ni-affinity column (right). Arrows indicate the positions of the SrTrx*o*1 protein on the gels. (**b**) Insulin reduction activities of recombinant SrTrx*o*1 at various temperatures. The extent of insulin level reduction by SrTrx*o*1 was determined at 15°C, 23°C and 30°C. RFU, relative fluorescence unit. Data are expressed as the mean ± SD (*n* = 3).

To clarify the functions of SrTrx*o*1 and SrTrx*o*2 as Trx enzymes, His-tagged recombinant proteins were produced in *E. coli.* The protein expression of SrTrx*o*1 but not SrTrx*o*2 was induced in the presence of IPTG, and the recombinant SrTrx*o*1 product could then be purified using the Ni-affinity column (Fig. 5a). An insulin reduction assay clearly showed that SrTrx*o*1 is functional in a temperature-dependent manner (Fig. 5b); the recombinant SrTrx*o*1 had a higher activity at 30°C compared with 23°C, whereas almost no activity was detectable at 15°C (Fig. 5b).

### SrTrxo1-mediated AOX reduction

**Fig. 6:**
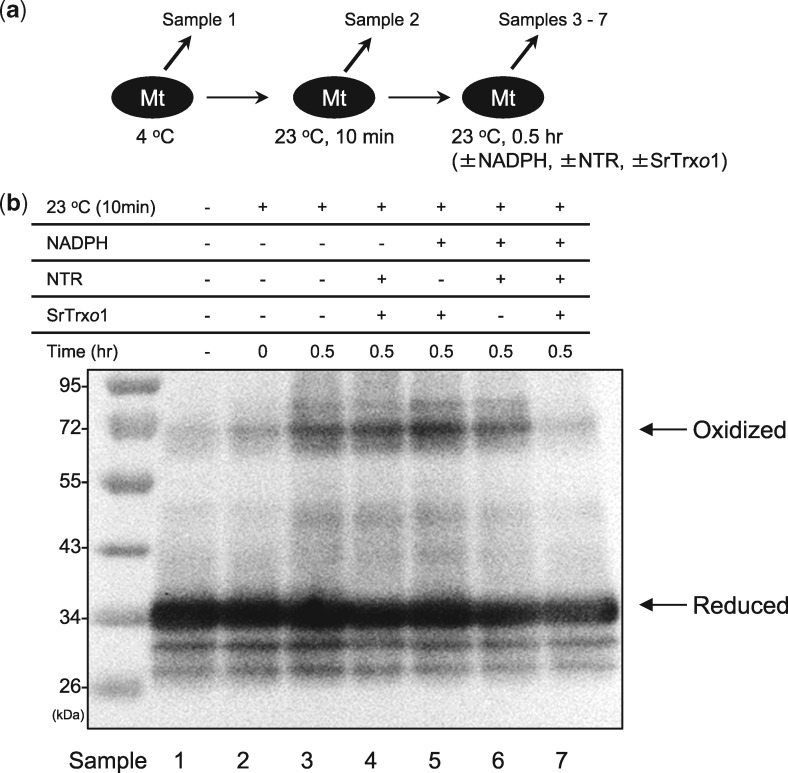
**Biochemical characterization of SrTrx*o*1-mediated AOX reduction in *S. renifolius*.** (**a**) Schematic representation of sample preparation for the western analysis of the redox status of SrAOX proteins. Mitochondrial membrane fractions placed at 4°C (sample 1) were incubated at 23°C for 10 min (sample 2) and for another 30 min either in the presence or absence of NADPH (5 mM), yeast NTR (3 µM) or SrTrx*o*1 (0.15 U/ml) (samples 3–7). (**b**) Western blotting analysis. Samples 1–7 were resolved by non-reducing SDS-PAGE and immunoblotted against polyclonal antibodies for AOX proteins. The oxidized and reduced forms of AOX proteins are indicated by arrows. Molecular size markers are also shown.

To determine whether the recombinant SrTrx*o*1 protein was capable of reducing oxidized AOX, Trx reduction assays were performed *in vitro* using recombinant SrTrx*o*1, yeast NTR and mitochondrial membrane fractions purified from the thermogenic florets of *S. renifolius* (Fig. 6a and b). Western blot analysis revealed that the SrAOX proteins were partially oxidized after incubation at 23°C for 30 min (Fig. 6b). Our data also showed that these oxidized SrAOX proteins were reduced only in the presence of NADPH, NTR and SrTrx*o*1 (Fig. 6b). These findings indicated that recombinant SrTrx*o*1 can reduce oxidized SrAOX proteins and that this process is mediated by NTR in thermogenic florets of *S. renifolius.*

## Discussion

### Two Trx o-type genes isolated from thermogenic spadix

We have successfully isolated two cDNAs for Trx *o* from the thermogenic spadix of *S. renifolius* and characterized their structure, gene expression patterns and function. Our current analyses of these Trx isoforms indicate that mitochondrial SrTrx*o*1-mediated AOX reduction plays a role in organ-specific thermogenesis in *S. renifolius.*

Multiple Trx family members have now been found in plants (14, 27), and the organ- and developmental stage-specific gene expression profiles have been documented for these Trx isoforms (28). In *Arabidopsis*, although the gene expression of *AtTrxo1* and *AtTrxo2* is ubiquitous, a higher expression was observed in ovaries and unicellular pollen (28). Our present study has also found that *SrTrxo1* transcripts are more highly expressed in thermogenic florets that include ovaries and pollen (Fig. 4a). It is thus possible that SrTrx*o*1 proteins play a role in floral development and/or reproduction processes. Moreover, previous evidence indicates that the overexpression of PsTrx*o*1 in tobacco BY-2 cells inhibits the production of exogenous hydrogen peroxide (29), and that Trx *o* could have a protective function against oxidation stress in mitochondria (30). In *S. renifolius*, thermogenic florets exhibit extremely high respiration rates (31) and an increase in ROS production from the ETC pathway (30) could potentially therefore oxidize reduced AOX proteins (active form) to an oxidized dimer (inactive form). Hence, the higher expression of *SrTrxo1* in the thermogenic florets found in our present experiments could significantly contribute to the continuous reduction of SrAOX proteins during thermogenesis in *S. renifolius.* The spathes of *S. renifolius* also accumulated a higher level of *SrTrxo1* transcripts than the piths or leaves (Fig. 4a). Because the non-thermogenic spathe has the largest surface area exposed to the air and can therefore often drop to freezing temperatures, we speculate that the higher expression of *SrTrxo1* in this tissue may reveal an uncharacterized role for this enzyme in a cold resistance response in *S. renifolius.*

### Amino acid sequences of SrTrxo1 and SrTrxo2

Although SrTrx*o*1 and SrTrx*o*2 possess several amino acid residues that are conserved among the Trx family proteins, SrTrx*o*2 specifically lacked the region that corresponds to the α3-helix of SrTrx*o*1 (Fig. 1a). The genomic sequencing of *S. renifolius* has not yet been completed but we speculate that SrTrx*o*2, a shorter SrTrx*o* protein isoform, is a splicing variant in *S. renifolius.* In this regard, it has been reported that *Trx* splicing variant lacking exons 2 and 3 is substantially expressed in human cancer cells and that this alternatively spliced isoform was not expressed as a stable protein (32). Intriguingly, the RGI motif of the α3-helix in *E. coli* Trx, which is lacking in SrTrx*o*2, contributes to the flexibility of the conformational changes in reduced Trx proteins (33). Because recombinant SrTrx*o*2 proteins were not expressed in our study (Fig. 5a), it could be postulated that endogenous SrTrx*o*2 proteins in cells are unstable. The expression of SrTrx*o*2 proteins in *S. renifolius* would then be expected to be low, irrespective of the transcript expression level. It should be noted here also that Trx *o* molecules lacking the α3-helix region have also been found in thermogenic *N. nucifera* (34) (Fig. 2). The use of alternative spliced transcripts, which do not produce any significant gene product, could be a regulatory mechanism by which actual wild-type mRNA levels for translation are repressed by competition (35). We thus speculate that the shorter isoforms of *Trx o* found in *S. renifolius* and *N. nucifera* may be involved in the regulation of *Trx o* gene expression in these thermogenic plants*.* Further studies are necessary to fully understand the physiological role of Trx *o* isoforms that specifically lack the α3-helix domain.

### Reduction of SrAOX by Trx system

The spadices of *S. renifolius* maintain their temperatures at around 23°C even when the ambient temperature drops below freezing (2), and the mitochondrial SrAOX proteins in thermogenic spadix of *S. renifolius* have been found in their reduced active form irrespective of external temperatures (5). However, as we found in our present study that the AOX proteins in mitochondrial membrane fractions placed at 23°C were partially oxidized, it is conceivable that biochemical regulatory systems for AOX reduction are active with a continuous supply of reducing equivalents. In addition, because yeast NTR-mediated SrTrx*o*1 was found to reduce oxidized SrAOX proteins in our heterogeneous assay system (Fig. 6), it is likely that SrTrx*o*1, identified in the present study, functions in the thermogenic cells in combination with a yet unidentified SrNTR located in the mitochondrial matrix of *S. renifolius.* In this case, a continuous generation and supply of NADPH as a coenzyme for SrNTR is necessary to fully activate the SrTrx*o*1 in mitochondria.

The origin of the NADPH then arises as a principal question as it has been shown that plant mitochondria do not contain an energy-dependent transhydrogenase that can maintain a reduced NADP pool by transfer of reducing equivalents from NADH to NADP^+^ (36). Unlike mammalian mitochondria, the synthesis of NADPH in plant mitochondria has been shown to be either from NADH by an NADH kinase or via an NADP-specific isocitrate dehydrogenase (ICDH), which is separate from the NAD-specific ICDH in the TCA cycle (37, 38). It should be noted here that we reported in our recent study that NADPH is abundantly produced by NADP^+^- ICDH in the mitochondria purified from thermogenic florets of *S. renifolius* (19). It is thus likely that production of NADPH mediated by ICDH, which is a characteristic process in mitochondria from the thermogenic florets of *S. renifolius*, plays an important role in continuous reduction of SrAOX via SrTrx*o*1 in this plant. It is interesting to note in this context that a functional NAD^+^-ICDH forms a heterodimer that is composed of regulatory and catalytic subunits, and that Trx *o* serves to convert oxidized regulatory subunits to their reduced form to activate the ICDH complex in *Arabidopsis* (39). Although it is not known whether NADP^+^-ICDH in thermogenic *S. renifolius* is comparably susceptible to oxidation stress and is a target for Trx *o* as found in *Arabidopsis* NAD^+^-ICDH, it is possible that the SrTrx*o*1 identified in this study regulates other enzymes including NAD^+^-ICDH that function in mitochondrial metabolisms in *S. renifolius.*

**Fig. 7:**
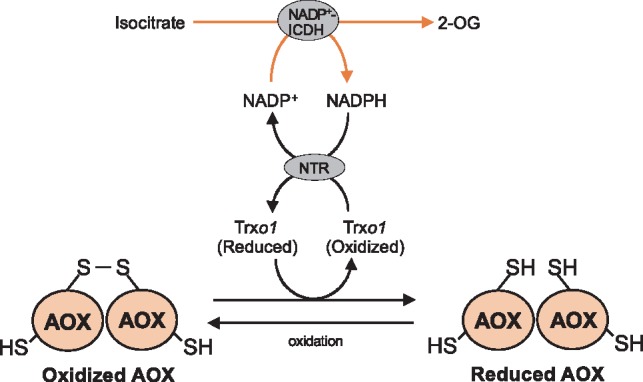
**Proposed model for the Trx *o*-mediated reduction of mitochondrial AOX in thermogenic spadices of *S. renifolius*.** NADP^+^ is reduced to NADPH via mitochondrial NADP^+^-ICDH, which catalyses isocitrate to 2-OG. Intra-mitochondrially produced NADPH is used for the NTR-mediated reduction of Trx*o*1, which reduces the intermolecular disulphide bond (Cys-1) of oxidized AOX proteins. 2-OG, 2-oxoglutarate; ICDH, isocitrate dehydrogenase; NTR, NADPH-dependent Trx reductase.

As summarized in Fig. 7, our current molecular and biochemical analyses suggest that the redox status of SrAOX proteins in thermogenic florets of *S. renifolius* is regulated by a Trx system composed of SrTrx*o*1 and NTR that consumes NADPH supplied by mitochondrial respiration mediated by NADP^+^-ICDH. To the best of our knowledge, our present study is the first to report the significance of the mitochondrial Trx system in regulating the redox status of AOX proteins in thermogenic plants. Further studies that are focused on the functions and target proteins of Trx*o*1 in other thermogenic plants will help to elucidate the more general mechanisms by which Trx*o*1 functions in metabolic heat-production in plants.

## Supplementary Material

Supplementary Table S1

Supplementary Table S2

## Abbreviations

2-OG: 2-oxoglutarate
AOX: alternative oxidase
BCA: bicinchoninic acid
ETC: electron transport chain
ICDH: isocitrate dehydrogenase
IPTG: isopropyl-β-d-thiogalactopyranoside
NTR: NADPH-dependent thioredoxin reductase
ORF: open reading frame
qRT-PCR: quantitative RT-PCR
Trx: thioredoxin
