# Supplementary material for: Thioredoxin o-mediated reduction of mitochondrial alternative oxidase in the thermogenic skunk cabbage Symplocarpus renifolius
Source: J Biochem. 2018 Oct 5;165(1):57–65. doi: 10.1093/jb/mvy082 (PMC6299270; doi:10.1093/jb/mvy082)
Supplement: Supplementary Table S1 [file mvy082_table_s1.pdf]

Table S1 DNA sequences of the primers used in this study.

Primer	Sequence 5' - 3'	Usage
Trx *o* forward (F)	TGAGTCAGAAATTCCCACATGTCACTAC	Partial cDNA cloning
Trx *o* reverse (R)	TAGTCCTTCTTGTAAAGATCTTCCATGGTG	Partial cDNA cloning
Trx *o* R1	GAACTTGAGATTAGGCACAGAGTAGACC	5'-RACE cloning
Trx *o* R2	GGATTTGCACCAATCACTTGAGCAGC	5'-RACE cloning
Trx *o* F1	TCCCACATGTCACTACATACAAGATCG	3'-RACE cloning
Trx *o* F2	TGATAAGGAGGGTCTTGAAAGCAAG	3'-RACE cloning
Trx *o* F3	GGTCTACTCTGTGCCTAATCTCAAGTTC	3'-RACE cloning
SrTrx *o* F1	CGGGGGCATCTGAAAGCAAG	Full length cDNA cloning
SrTrx *o* F2	GAAAGCAAGTACCCTCCCCCG	Full length cDNA cloning
SrTrx *o* F3	GAGTCGCCACCTCTCCGAAA	Full length cDNA cloning
SrTrx *o* R1	TCTTCTCCTCCCAATCCCTACGC	Full length cDNA cloning
realtimeSrTrx*o*1 F	CGATATTGATAAGGAGGGTCTTG	qRT-PCR
realtimeSrTrx*o*2 F	GATCGATATTGATAAGCCTAATCTC	qRT-PCR
realtimeSrTrx*o* R	AATTGGTTCTACTGCTTGTCG	qRT-PCR
realtimeSrAOX F	AAGGAGATCGACAACGGGACCATC	qRT-PCR
realtimeSrAOX R	CTGGTAATGGATGTCCGAGGCAAAG	qRT-PCR
realtimeSrEF1α F	AGCATTGTGGTCATTGG	qRT-PCR
realtimeSrEF1α R	CTCTTGTTCATCTCAGCAG	qRT-PCR
