# Supplementary material for: Thioredoxin o-mediated reduction of mitochondrial alternative oxidase in the thermogenic skunk cabbage Symplocarpus renifolius
Source: J Biochem. 2018 Oct 5;165(1):57–65. doi: 10.1093/jb/mvy082 (PMC6299270; doi:10.1093/jb/mvy082)
Supplement: Supplementary Table S2 [file mvy082_table_s2.pdf]

Table S2 Accession numbers of the Trx family proteins used in the phylogenetic analysis.

Plant species	Definition of thioredoxin family protein in this paper	Accession number
Arabidopsis thaliana	AtTrx*f*1	NP_186922.1
	AtTrx*f*2	NP_197144.1
	AtTrx*h*1	NP_190672.1
	AtTrx*h*2	NP_198811.1
	AtTrx*m*1	NP_849585.1
	AtTrx*m*2	NP_192261.1
	AtTrx*o*1	NP_181046.1
	AtTrx*o*2	NP_564371.1
	AtTrx*x*	NP_564566.1
	AtTrx*y*1	NP_177802.2
	AtTrx*y*2	NP_175021.2
	AtTrx*z*	NP_187329.1
Brachypodium distachyon	BdTrx*o*	XP_003563361.1
Nelumbo nucifera	NnTrx*f*	XP_010246376.1
	NnTrx*h* X1	XP_010254672.1
	NnTrx*h* X2	XP_010275525.1
	NnTrx*m*	XP_010270409.1
	NnTrx*o*2 X1	XP_010260626.1
	NnTrx*o*2 X2	XP_010260627.1
	NnTrx*o*2 X3	XP_010260628.1
	NnTrx*x*	XP_010249669.1
	NnTrx*y*1	XP_010241685.1
	NnTrx*z*	XP_010269422.1
Oryza sativa	OsTrx*f*	XP_015616604.1
	OsTrx*h*	BAB20886.1
	OsTrx*m*	XP_015620324.1
	OsTrx*o*	XP_015641364.1
	OsTrx*x*	XP_015636655.1
	OsTrx*y*	EEE56056.1
	OsTrx*z*	XP_015648902.1
Populus euphratica	PeTrx*o*1 X2	XP_011035473.1
	PeTrx*o*1 X3	XP_011035475.1
	PeTrx*o*2 X1	XP_011035472.1
Symplocarpus renifolius	SrTrx*o*1	BAU24795.1
	SrTrx*o*2	BAU24796.1
Vitis vinifera	VvTrx*o*2	XP_002275152.1
Zea mays	ZmTrx*o*	NP_001149559.1
